# Synthesis, X-ray Structure and Biological Studies of New Self-Assembled Cu(II) Complexes Derived from *s*-Triazine Schiff Base Ligand

**DOI:** 10.3390/molecules27092989

**Published:** 2022-05-06

**Authors:** Tarek E. Khalil, Kholood A. Dahlous, Saied M. Soliman, Nessma A. Khalil, Ayman El-Faham, Ali El-Dissouky

**Affiliations:** 1 Department of Chemistry, Faculty of Science, Alexandria University, P.O. Box 426, Ibrahimia, Alexandria 21321, Egypt; nessma_1988@outlook.com (N.A.K.); aymanel_faham@hotmail.com (A.E.-F.); alieldissouky@alexu.edu.eg (A.E.-D.); 2 Department of Chemistry, College of Science, King Saud University, P.O. Box 2455, Riyadh 11451, Saudi Arabia

**Keywords:** *s*-Triazine, Cu(II) complexes, antimicrobial, antioxidant, anticancer, lung carcinoma

## Abstract

The two ligands 2-(1-(2-(4,6-dimorpholino-1,3,5-triazin-2-yl)hydrazono)ethyl)aniline (**DMAT**) and 2-(1-(2-(4,6-dimorpholino-1,3,5-triazin-2-yl)hydrazono)ethyl)phenol (**DMOHT**) were used to synthesize three heteroleptic Cu(II) complexes via a self-assembly technique. The structure of the newly synthesized complexes was characterized using elemental analysis, FTIR and X-ray photoelectron spectroscopy (XPS) to be [Cu(DMAT)(H_2_O)(NO_3_)]NO_3_.C_2_H_5_OH (**1**), [Cu(DMOT)(CH_3_COO)] (**2**) and [Cu(DMOT)(NO_3_)] (**3**). X-ray single-crystal structure of complex **1** revealed a hexa-coordinated Cu(II) ion with one **DMAT** as a neutral tridentate *NNN*-chelate, one bidentate nitrate group and one water molecule. In the case of complex **2**, the Cu(II) is tetra-coordinated with one **DMOT** as an anionic tridentate *NNO*-chelate and one monodentate acetate group. The antimicrobial, antioxidant and anticancer activities of the studied compounds were examined. Complex **1** had the best anticancer activity against the lung carcinoma A-549 cell line (IC_50_ = 5.94 ± 0.58 µM) when compared to *cis*-platin (25.01 ±2.29 µM). The selectivity index (SI) of complex **1** was the highest (6.34) when compared with the free ligands (1.3–1.8), and complexes **2** (0.72) and **3** (2.97). The results suggested that, among those compounds studied, complex **1** is the most promising anticancer agent against the lung carcinoma A-549 cell line. In addition, complex **1** had the highest antioxidant activity (IC_50_ = 13.34 ± 0.58 µg/mL) which was found to be comparable to the standard ascorbic acid (IC_50_ = 10.62 ± 0.84 µg/mL). Additionally, complex **2** showedbroad-spectrum antimicrobial action against the microbes studied. The results revealed it to possess the strongest action of all the three complexes against *B. subtilis*. The MIC values found are 39.06, 39.06 and 78.125 μg/mL for complexes **1**–**3**, respectively.

## 1. Introduction

Hydrazones have been considered as initiating compounds for most of synthetic chemistry, offering multifunctional benefits to various fields, such as industrial and pharmacological applications [1,2,3,4,5,6,7]. They have played an essential role in analytical and medicinal chemistry, as well as in coordination chemistry as polydentate ligands because of their versatile chelating capacity with most transition metals [8,9,10,11,12]. On the other hand, Schiff base hydrazone derivatives have attracted great attention because of their valuable applications as heterogeneous catalysts, molecular switching materials and analytical reagents, and for their use in forming covalent organic frameworks [13,14,15]. Additionally, they have been demonstrated to be bioactive compounds that can be widely used for the treatment of many diseases caused by some types of bacteria and viruses; they inhibit the replication of these viruses or bacteria inside living cells. Hydrazones have shown encouraging results as antidepressants, antimalarial, anticancer, anti-inflammatory, and antioxidant agents as well [16,17,18,19,20].

On the other hand, *s*-Triazines play integral roles as a organic ligands in coordination chemistry [21,22,23,24,25,26,27] and as organic building units in supramolecular chemistry [28,29,30]. Another advantage is the synthetic versatility of *s*-Triazine derivatives which comes from the simple and inexpensive way they can be prepared from cyanuric chloride [31,32,33]. *s*-Triazines exhibit a wide range of industrial and agricultural uses, including in the preparation of resins, dyes for textiles, removal agents for sulfide and in herbicides [34,35]. In addition, they have an essential position in medicinal chemistry since they are used in the manufacturing of many drugs with various pharmacological activities [36,37]. Additionally, Schiff bases containing *s*-Triazine have a wide range of biological activities, especially as promising anticancer agents [38,39,40,41]. Over and above these, Cu(II) complexes with *s*-Triazine hydrazone derivatives have interesting magnetic and catalytic applications [42,43,44,45,46], as they play a catalytic role in numerous chemical and biological processes, concurrently performing an essential function in the immune system, nutrient metabolism, as well as serving as a metal cofactor bound to protein in the living body. Although copper is a trace metal, its numerous complexes are now gaining attention because of their prospective uses as antiviral, antibacterial, anti-inflammatory drugs with decreased unwanted effects and as anticancer medicines [47,48,49,50,51,52,53,54].

**Figure 1:**
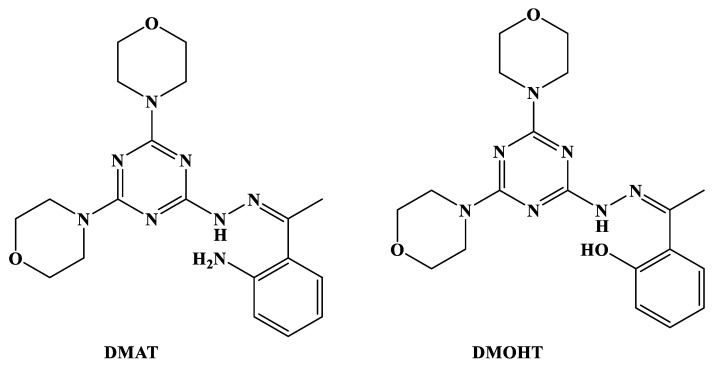
Structures of the studied ligands.

Motivated by the facts outlined above, the current study focuses on the synthesis and spectroscopic characterizations of three new Cu(II) complexes with the 2-(hydrazino)-4,6-disubstituted-*s*-Triazine ligands shown in Figure 1. X-ray diffraction of single- crystal, Hirshfeld, and spectroscopic analyses were used to investigate the structural characteristics of the synthesized complexes. Furthermore, the antibacterial, antioxidant and anticancer activities of these complexes were studied and compared with free ligands.

## 2. Results and Discussion

### 2.1. Synthesis and Characterizations

**Scheme 1:**
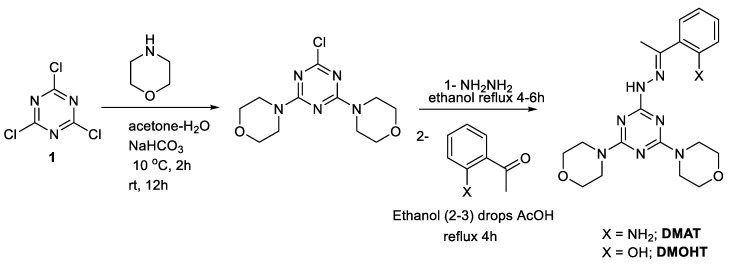
Synthesis of DMAT and DMOHT.

The two ligands **DMAT** and **DMOHT** were prepared following the reported method [55]. The first and second chlorine atoms of the 2,4,6-trichloro-1,3,5-triazine (cyanuric chloride) were replaced in one step by two morpholine. Then the third chlorine atom was replaced by hydrazine under reflux for 4–6 h (Scheme 1). Finally, the hydrazine derivatives were condensed with 2-amino-/or 2- hydroxy-acetophenone in the presence of a few drops of acetic acid and ethanol as a solvent in order to give the target products in high yields and with high purities, as observed from their NMR spectra, which are in good agreement with the reported data (Figures S1 and S2; Supplementary material).

**Figure 2:**
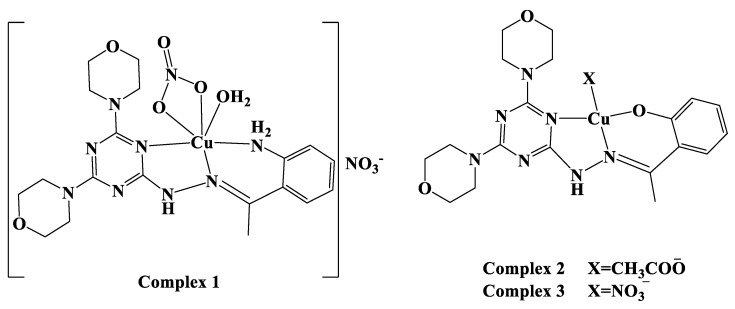
Structures of the synthesized Cu(II) complexes.

The three new Cu(II) complexes were synthesized using a self-assembly technique. The newly synthesized complexes were characterized using FTIR, elemental analysis and X-ray photoelectron spectroscopy (XPS). The FTIR spectra of the studied complexes are shown in Figures S3 and S4 (Supplementary material). The schematic presentation for the proposed structures of the studied complexes is presented in Figure 2. The structures of two of the newly synthesized complexes were further confirmed using single-crystal X-ray diffraction. The results were found to be in accordance with the proposed structures based on the analytical and spectral analyses.

### 2.2. X-ray Structure

#### 2.2.1. Structure Description of Complex 1

**Figure 3:**
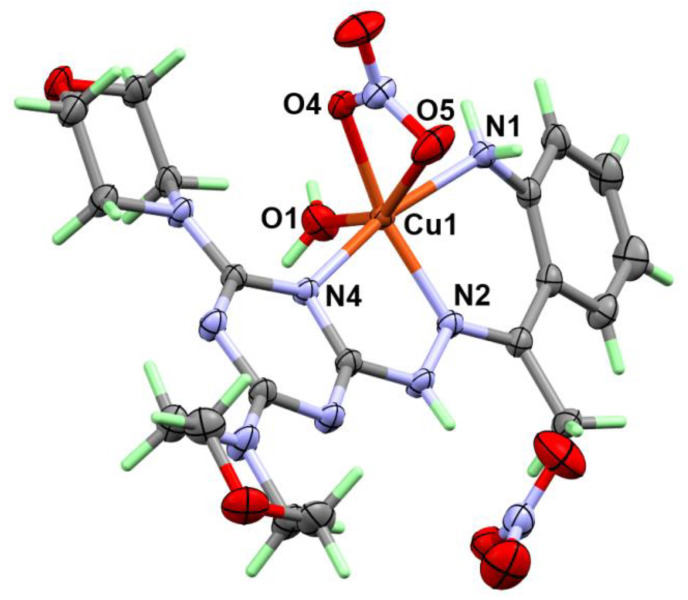
Structure and atomic numbering of complex **1**. The disordered ethanol molecule was omitted for better clarity.

The X-ray structure together with the atomic numbering of the [Cu(DMAT)(H_2_O)(NO_3_)]NO_3_.C_2_H_5_OH complex (**1**) are shown in Figure 3. A list of the most important geometric parameters is given in Table 1. Complex **1** crystallized in the triclinic crystal system and P-1 space group and Z = 2 (Table S1; Supplementary material). The Cu(II) was coordinated with one neutral **DMAT** ligand as a tridentate *NNN*-chelate, with one water molecule and a nitrate group leading to the cationic coordination sphere [Cu(DMAT)(H_2_O)(NO_3_)]^+^. The outer sphere comprised one nitrate counter anion and one highly disordered ethanol molecule. The coordination centers of the organic ligand were the N-sites from the amino group, hydrazone and triazine moieties. All of the three Cu-N distances recorded are marginally different (Table 1). The N1-Cu1-N2 (88.17(9)˚) and the N2-Cu1-N4 (82.26(8)˚) bite angles of the **DMAT** ligand led to the stable six membered and five membered chelate rings, respectively. The coordination environment of the Cu(II) was completed by two strong interactions with one oxygen atom from the coordinated nitrate (Cu1-O4; 2.006(2) Å) and another oxygen atom from a water molecule (Cu1-O1; 2.185(2) Å) augmented with an extra weak interaction with an oxygen atom from the same nitrate group (Cu1-O5; 2.651(2) Å). Hence, the coordination number of Cu(II) found was six, and the nitrate group acting as a bidentate ligand where the bite angle of the bidentate nitrate group was only 53.28(8)˚.

**Figure 4:**
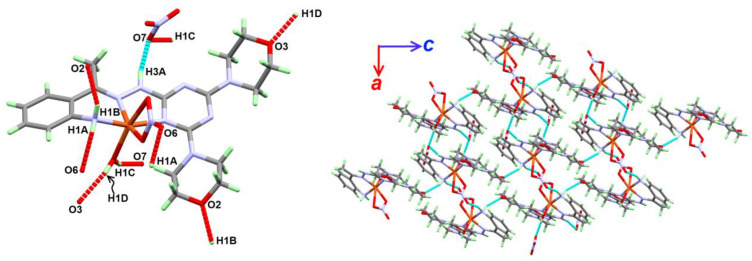
The hydrogen bond contacts (**left**) and packing along the *ac* plane (**right**).

The molecular packing of complex **1** was controlled by strong N-H…O and O-H…O hydrogen bonding interactions (Table 2). The presentation of these contacts is shown in the left part of Figure 4, while the right part of the same figure presents the packing along the *ac* plane. The N-H of the amino groups and the O-H of the water molecule are the hydrogen bond donors, while the hydrogen bond acceptor sites are the oxygen atoms that are either from the nitrate or morpholine moieties.

**Figure 5:**
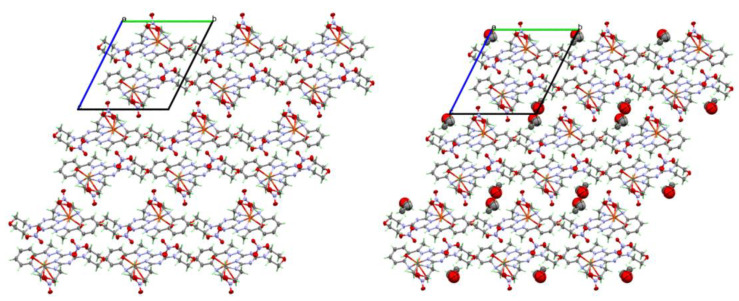
The packing structures of complex **1** with (**left**) and without (**right**) the disordered ethanol molecule.

It is worth noting that the crystallized ethanol molecules occupy the spaces among the complex units and are located in between the coordinated and ionic nitrate anions along the *b* and *c* directions, which further aid in the supramolecular structure of the studied complex. For a better understanding, the packing structures of the complex units with and without the solvent molecule are depicted in Figure 5. Unfortunately, the corresponding hydrogen bond parameters could not be correctly determined because of the presence of strong disorder in the ethanol molecule.

#### 2.2.2. Structure Description of Complex 2

**Figure 6:**
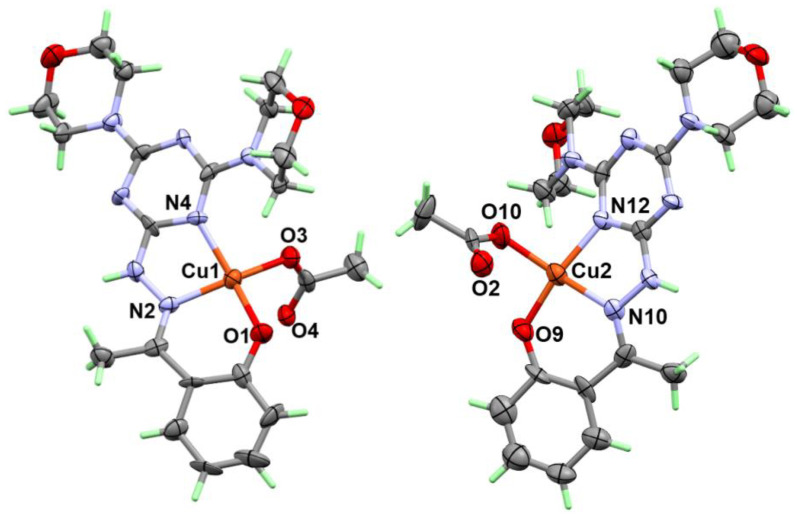
Structure and atom numbering of the coordination sphere of complex **2**.

The X-ray structure of complex **2** satisfactorily revealed the tetra-coordination environment of the Cu(II). This complex crystallized in the more symmetric monoclinic crystal system and the P2_1_/n space group while the unit cell comprised four molecules (Z = 4) (Table S1; Supplementary material). The structure of the coordination sphere of complex **2** could be simply described as [Cu(DMOT)(CH_3_COO)]. In this neutral complex, the organic ligand acted as a mononegative tridentate *NNO*-chelate, where the hydroxyl group was deprotonated during the course of the preparation owing to the presence of the relatively strong basic acetate anion (Figure 6).

**Figure 7:**
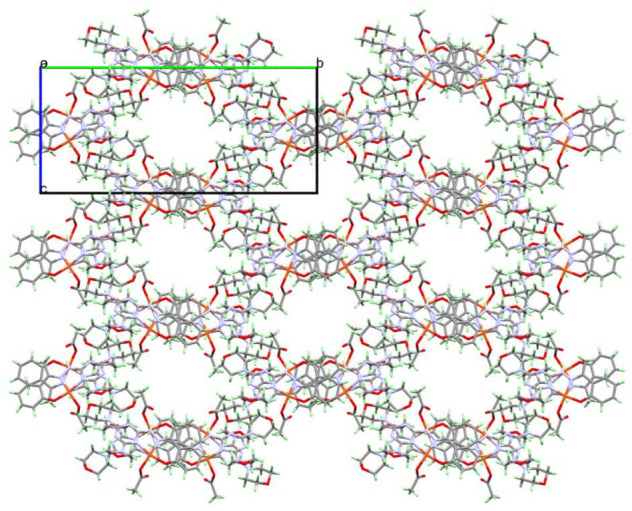
Packing scheme of the complex units of **2**. In such tubular structures, these spaces are occupied by the highly disordered solvent (water) molecules, where their protons could not be refined properly.

There are two molecular units that can be described as an asymmetric formula, where the two Cu(II) centers have similar coordination spheres and differ very little in Cu-N and Cu-O distances (Table 3). It is clear from the table below that the Cu-N_(hydrazone)_ bonds are slightly shorter than the Cu-N_(*s*-Triazine)_ in both complexes. Generally, the Cu-N distances are shorter in complex **2** when compared to those found in complex **1**. The tetra-coordination environment of the Cu(II) is completed by one coordinated acetate ion as a monodentate ligand. The Cu-O distances are found to be 1.905(7) Å (Cu1-O3) and 1.902(8) Å (Cu2-O10), which are generally longer than the distances between the Cu(II) and the phenolic oxygen atom. The distances between the Cu1 and Cu2 centers to the O4 and O2 atoms of the acetate anion were found to be 2.961(6) and 2.953(7) Å, respectively, both of which are too long to be bonds. Hence, the acetate anion in both units could be described as a monodentate ligand. The coordination geometry around Cu(II) could be described as a distorted square planar; the packing scheme is shown in Figure 7.

### 2.3. Analysis of Molecular Packing

**Figure 8:**
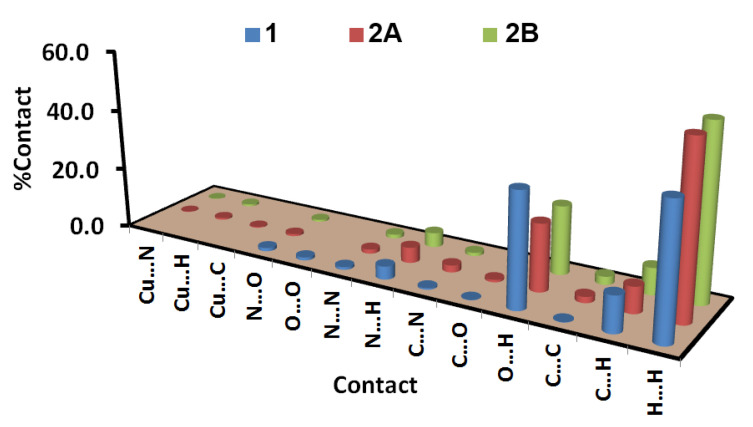
The intermolecular interactions in complexes **1** and **2**.

**Figure 9:**
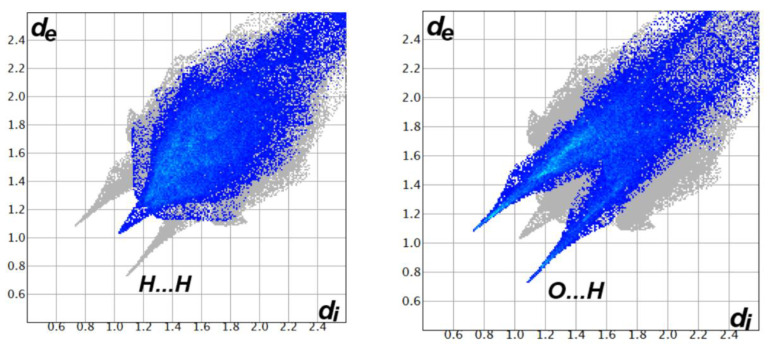
Decomposed fingerprint plots in complex **1**.

**Figure 10:**
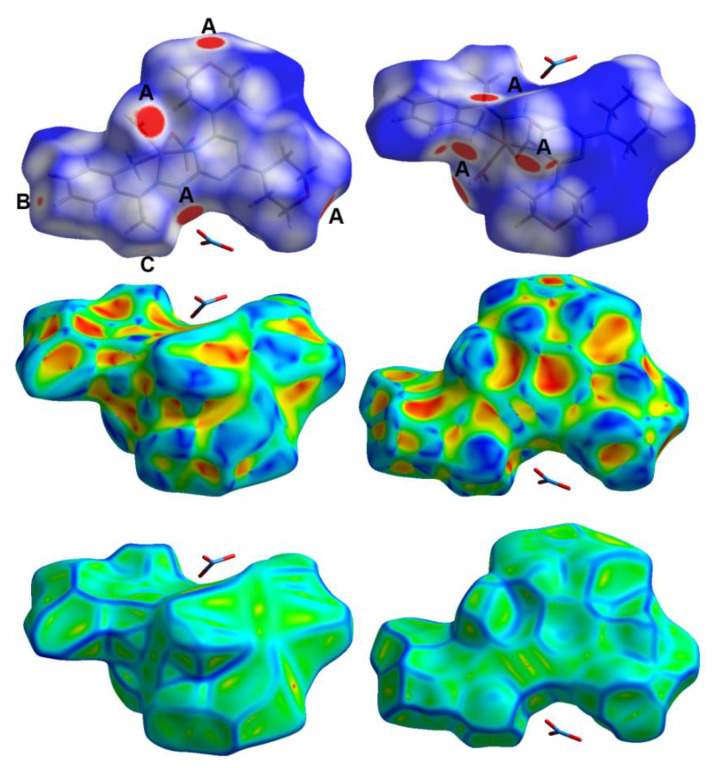
Hirshfeld maps of complex **1**. The most important interactions are (**A**) O…H, (**B**) H…H and (**C**) C…C.

Hirshfeld maps are characteristic of each crystal, and our analysis of these maps gave us quite a clear idea about the intermolecular interactions included in the molecular packing within a crystal. The distribution of the different contacts for complexes **1** and **2** are presented in Figure 8. In the case of complex **1**, the percentages of the O…H, H…H and H…C contacts are the highest. Their percentages are 37.2, 43.0 and 11.8%, respectively. Analysis of the fingerprint plot of complex **1** revealed that both O…H and H…H are short contacts, as these interactions appeared as sharp spikes (Figure 9). The regions included in the H...H and O…H interactions are labelled in the d_norm_ map as regions **A** and **B**, respectively (Figure 10). These regions are marked with a red color, further indicating interactions shorter than the vdWs radii sum of the interacting atoms.

The shortest O…H interactions are O7…H1C and O3…H1D, where the hydrogen-acceptor distances are 1.841 and 1.820 Å, respectively. Generally, there are quite a large number of O…H interactions, with hydrogen-acceptor distances ranging from 1.820 Å to 2.601 Å (O5…H14A). Additionally, there are two other short interactions which belong to H4…H4 (2.074 Å) and C11…C11 (3.350 Å)—interactions which are considered important in the molecular packing of **1**. A summary of short contacts is listed in Table 4.

**Figure 11:**
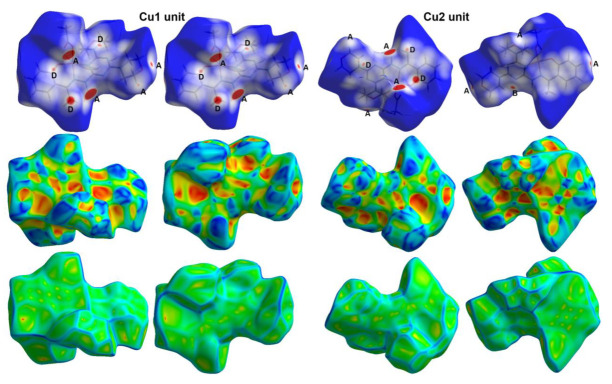
Hirshfeld maps of complex **2**. The most important interactions are (**A**) O…H, (**B**) N…H, (**C**) C…C and (**D**) H…C.

**Figure 12:**
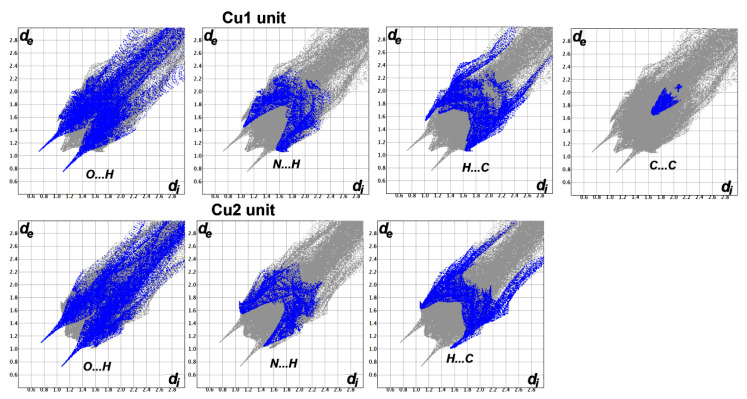
Decomposed fingerprint plots in complex **2**.

For complex **2**, there are also many short interactions that were detected as red spots in the d_norm_ map. In this compound, there are two molecules used as an asymmetric formula; hence, there are two results for the Hirshfeld analysis in this case (Figure 11). Similar to complex **1**, the H…H, O…H and H…C contacts are the most dominant. Their percentages are in the ranges of 56.1–56.6, 22.0–22.5 and 8.6–8.8%, respectively. There are many other contacts that are depicted in Figure 8, but their percentages were relatively small. Among the whole of the interactions which occurred in the crystal of complex **2**, the O…H, N…H, H…C and C…C contacts were considered short and appeared as red regions in the d_norm_ map (Figure 11). The fingerprint plots shown in Figure 12 amply indicate the importance of these contacts. O2…H3 (1.802 Å), N14…H14B (2.490 Å), C22…H11B (2.547 Å) and C1…C7 (3.332 Å) are the shortest of these interactions (Table 4).

### 2.4. XPS Study

XPS analysis is a potent, surface-sensitive method used for checking the chemical composition and purity of atoms, and it can be used to determine the core electron binding energy of atoms within a surface layer up to 10 nm. The correlation between the binding energy (BE) and the atomic potential provides information concerning atoms’ oxidation states [56,57,58]. The wide-ranging scan XPS spectra of the free ligands (**DMAT**, **DMOHT**) and their copper (II) complexes are displayed in Figure S5 (Supplementary material) and the individual contributions within the high-resolution spectra for the different XPS parameters that were collected at 1253.6 eV for Cu(2p3/2), Cu(2p1/2), C1s, O1s and N1 are given in Tables S2 and S3 (Supplementary material). The XPS spectrum for the free ligand (**DMAT**) displays three peaks resulting from the binding energies (BEs) of C1s, N1s and O1s at 286.17, 399.75 and 532.94 eV, respectively. The high-resolution C1s spectra are characterized by contributions at 287.05, 284.71 and 289.37 eV in **DMAT** arising from the C–H, C = C (sp^2^ bonded carbons), C–C (sp^3^ bonded carbons) and C–N moieties. The de-convolution of the N1s peak shows two peaks, one at 399.45 and another at 398.01 eV, which can be attributed to triazine C = N and NH confirming the structure of the ligand. The full scan spectrum of complex **1** exhibits four peaks corresponding to the BEs of C1s, N1s, O1s and Cu2p at 287.22, 400.85, 533.74 and 935.42 eV, respectively (Figure S5; Supplementary material). Cu2p displayed a satellite structure with two characteristic peaks—Cu2p3/2 and Cu2p1/2 at 934.28 and 954.23 eV, respectively, with a binding energy difference Δ(BE) = 19.95 eV) characteristic in accordance with the data reported for the existence of the Cu(II) oxidation state [59].

The XPS spectrum for the free ligand (**DMOHT**) displays three peaks resulting from the binding energies (BEs) of C1s (286.24 eV), N1s (399.94 eV) and O1s (533.13 eV) because of C-C (sp^3^), C-H, and C=C (sp^2^), as well as H-N, C=N and O-C, respectively. The spectra of complex **2** exhibited four peaks corresponding to the BEs of C1s (286.07 eV), N1s (399.87 eV), O1s (532.82 eV) and Cu2p (934.44 eV). The respective values for complex **3** are 285.91, 407.85, 533.16 and 935.44 eV. Cu2p displayed a satellite structure with two characteristic peaks, Cu2p3/2 (932.25 eV) and Cu2p1/2 (952.14 eV), for complex **2**. The respective values for complex **3** are 934.72 and 954.71 eV. As a result, the binding energy difference (ΔBE) is 19.89 eV for complex **2** and 19.99 eV for complex **3**. This finding is in agreement with the data reported for the Cu(II) oxidation state [59].

### 2.5. FTIR Spectra

The FTIR spectra of the free ligands and their Cu(II) complexes are presented in Figures S3 and S4 (Supplementary material). For complex **1**, a new band was observed at 3444 cm^−1^ corresponding to the overlapping ν(OH) modes for the coordinated water and crystal ethanol molecules, while the ν(N-H) mode of the hydrazone appeared at the same frequency of 3277 cm^−1^ in **DMAT** and **1**. The ν(N-H) modes of the amino group appeared at the lower frequencies of 3167 and 3112 cm^−1^ in the complex compared to 3434 and 3390 cm^−1^ in the free ligand, respectively. In addition, the FTIR spectrum of complex **1** showed a sharp spectral band at 1620 cm^−1^ which can probably be assigned to the ν(C==N) modes while appearing in the free ligand in the spectral range of 1577–1611 cm^−1^, indicating the coordination of the Cu(II) ion via the triazine and the azomethine nitrogen atoms. The infrared vibrational band appeared at 1386 cm^−1^ in complex **1**, which does not appear in the free ligand and could be attributed to the ν(N-O) stretches of the nitrate group [60].

The FTIR spectra of the free **DMOHT** ligand demonstrated the ν(OH) and ν(NH) modes at 3440 and 3314 cm^−1^, respectively, while the ν(C=N) modes were detected at 1579–1611 cm^−1^. The ν(C=N) modes were slightly shifted to 1614–1570 and 1608–1568 cm^−1^, respectively. In complex **3**, the ν(N-O) stretches of the nitrate group appeared at 1384 cm^−1^. On the other hand, two characteristic bands at 1569 and 1362 resulted from the asymmetric ν(COO) and symmetric ν(COO) stretching frequencies in complex **2**. The difference [Δ*ν* = *ν*(COO)asym − *ν*(COO)sym = 207 cm^−1^] indicates a monodentate coordination mode in the acetato group.

### 2.6. Biological Studies

#### 2.6.1. Antimicrobial Activity

The antimicrobial activity of the free ligands and the corresponding Cu(II) complexes was determined against different categories of microbes (Table 5). All inhibition zones were determined at 10 mg/mL of the tested compounds. The results indicated that both ligands were not active at the concentration of 10 mg/mL, while the corresponding Cu(II) complexes showed different antimicrobial actions against the tested microbes. Complex **2** showed broad antimicrobial activity against all tested microbes. The largest inhibition zones were found for *B. subtilis* (25 mm), which is very close to the standard Gentamycin (26 mm). The same complex also showed good antifungal activity against *A. fumigatus* (15 mm) while the corresponding value for the standard Ketoconazole used as a control is 17 mm. Complex **1** evidenced antimicrobial activity against all microbes except *C. albicans*. The best result for this complex was found to be against *B. subtilis* (25 mm). On the other hand, complex **3** showed no antifungal activity against either *A. fumigatus* or *C. albicans*, while its antibacterial action remained evident. Likewise, the best result for this complex was found to be against *B. subtilis* (24 mm).

The antimicrobial activity levels of complexes **1–3** were further revealed by determining the Minimum Inhibitory Concentrations (MIC) in μg/mL. The results indicate the strongest actions for all three complexes were against *B. subtilis*. The MIC values in these cases were found to be 39.06, 39.06 and 78.125 μg/mL, respectively, while for Gentamycin the MIC value is 4.8 μg/mL. Regarding the level of antifungal activity, complex **2** exhibited the best results against *A. fumigatus* (625 μg/mL) while the control Ketoconazole has MIC of 156.25 μg/mL (Table 6).

#### 2.6.2. Antioxidant Activity

The studied Cu(II) complexes and the free ligands were assessed for their antioxidant activity levels using an α,α-diphenyl-β-picrylhydrazyl (DPPH) scavenging assay, and the results are given in Table 7. All compounds possessed some antioxidant activity. The results indicated better antioxidant activity for **DMAT** than for **DMOHT,** indicating the effect of the substituent at the phenyl moiety on the antioxidant activity level of the free ligand. The IC_50_ values were determined to be 17.83 ± 0.65 µg/mL and 48.2 ± 1.086 µg/mL, respectively. On the other hand, the Cu(II) complexes have improved levels of antioxidant activity compared to the free ligands. Complex **1** has an IC_50_ value of 13.34 ± 0.58 µg/mL, which is lower than that of the free **DMAT**. Additionally, complexes **2** and **3** have lower IC_50_ values of 20.93 ± 0.91 and 19.31 ± 0.73 µg/mL, respectively, compared to **DMOHT**. Hence, the Cu(II) complexes appear to be better candidates for antioxidant agents than the free ligands, and **1** is the best performing among the studied Cu(II) complexes when compared with the standard ascorbic acid (IC_50_ = 10.62 ± 0.84 µg/mL).

#### 2.6.3. Anticancer Activity

**Figure 13:**
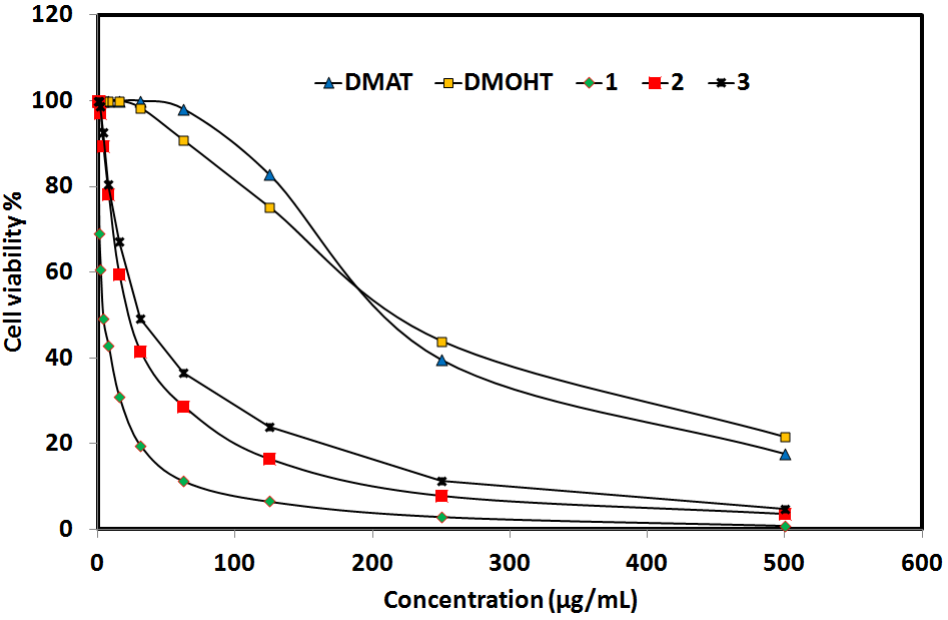
The anticancer activity of the studied compounds versus *Lung carcinoma*.

The studied compounds were examined for their anticancer activity against the lung carcinoma A-549 cell line. Detailed results are depicted in Tables S4–S8 and summarized graphically in Figure 13. The free ligands **DMAT** and **DMOHT** have large IC_50_ values of 551.48 ± 27.20 µM and 564.51 ± 30.94 µM, respectively, indicating a slightly better level of anticancer activity for **DMAT** than for **DMOHT**. In contrast, all the Cu(II) complexes showed good levels of anticancer activity as indicated by their lower IC_50_ values when compared to the free ligands. The IC_50_ value was the best for complex **1** (5.94 ± 0.58 µM) while the corresponding values for complexes **2** and **3** were 141.47 ± 3.24 µM and 58.34 ± 3.87 µM, respectively. Hence, the order of the anticancer activity for studied compounds is **1 > 3 > 2 >** **DMAT** > **DMOHT**. For *cis*-platin, the IC_50_ value is 25.01 ±2.29 µM, indicating that complex **1** had four times higher anticancer activity than the standard *cis*-platin.

**Figure 14:**
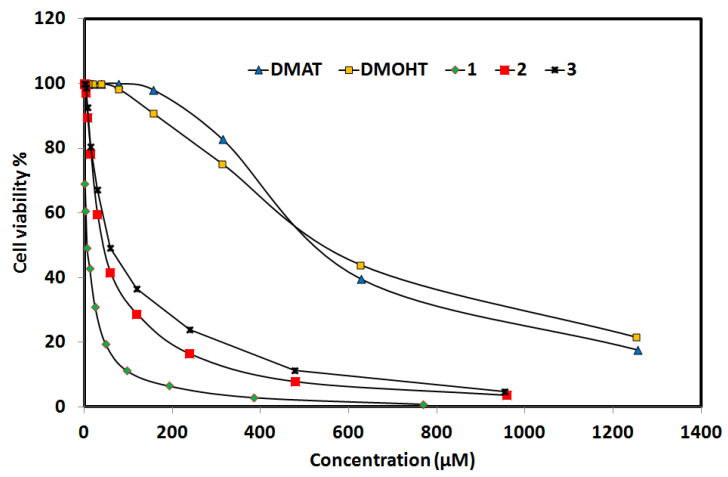
The cytotoxic activity of the studied compounds against normal human lung fibroblast MRC-5 cells.

Additionally, the degree of inhibitory cytotoxic activity against normal human lung fibroblast MRC-5 cells was determined and the results were collected in Tables S9–S13 and presented graphically in Figure 14. The CC_50_ values for indicating the degree of cytotoxic activity are 716.04 ± 49.36 µM and 999.90 ± 57.13 µM for the free **DMAT** and **DMOHT**, respectively. For complexes **1–3**, the CC_50_ values are 37.67 ± 2.97 µM, 101.47 ± 7.01 µM and 172.96 ± 11.98 µM, respectively. Based on these results, complex **1** shows the highest degree of cytotoxicity against the MRC-5 cell line. In this regard, the selectivity index (SI) was calculated as the ratio of cytotoxic effect on the normal cell line to the cytotoxic effect on the cancer cell line. The SI results revealed that complex **1** had the highest value (6.34) when compared with complexes **2** (0.72) and **3** (2.97). The SI values for the free ligands **DMAT** and **DMOHT** are 1.30 and 1.77, respectively. Hence, complex **1** is the best candidate for consideration as an anticancer agent against lung carcinoma (Table 8).

## 3. Materials and Methods

Fourier transform infrared (FTIR) spectra were recorded on a Nicolet 6700 spectrophotometer in the spectral range of 400–4000 cm^−1^ at a spectral resolution of 2 cm^−1^ and with 40 scans per sample. The powder samples were measured in KBr pellets. Further details regarding materials and methods are presented in the Supplementary material.

### 3.1. Synthesis of Ligands

The two ligands **DMAT** and **DMOHT** were prepared following our previously reported method described in Method S1 (Supplementary material) [55].

### 3.2. Syntheses of Cu(II) Complexes

All complexes were prepared using the self-assembly of the functional ligand shown in Figure 1 with the corresponding Cu(II) salt.

#### 3.2.1. Synthesis of Complex **1**

An ethanolic solution (10 mL) of **DMAT** (79.7 mg, 0.2 mmol) was added to 48.32 mg of Cu(NO_3_)_2_·3H_2_O in (5 mL) ethanol. The clear deep blue solution was left for slow evaporation at room temperature. After ten days, complex **1** was formed as dark blue crystals. **C_21_H_34_CuN_10_O_10_** (**1**; 650.12 g/mol): Yield; 83%. Anal. Calc. C, 38.80; H, 5.27; N, 21.55; Cu, 9.77%. Found: C, 38.58; H, 5.19; N, 21.41; Cu, 9.65%. IR (KBr, cm^−1^): 3444, 3277, 3167, 3112, 1620, 1550 and 1386.

#### 3.2.2. Synthesis of Complex **2**

A methanolic solution (10 mL) of **DMOHT** (79.9 mg, 0.2 mmol) was added to 24.5 mg of Cu(CH_3_COO)_2_ in (5 mL) methanol. The clear deep blue solution was left for slow evaporation at room temperature. After five days, complex **2** formed as dark blue crystals. **C_21_H_27_CuN_7_O_5_** (**2**; 521.03 g/mol): Yield; 79%. Anal. Calc. C, 48.41; H, 5.22; N, 18.82; Cu, 12.20%. Found: C, 48.17; H, 5.14; N, 18.68; Cu, 12.09%. IR (KBr, cm^−1^): 3437, 1614, 1570, 1508 and 1441.

#### 3.2.3. Synthesis of [Cu(DMOT)(NO_3_)] Complex **3**

Synthesis of this complex was performed the same way as described in **1**. In this case, several trials to obtain good quality crystals suitable for single-crystal structure measurements failed. **C_19_H_24_CuN_8_O_6_** (**3**; 523.99 g/mol): Yield; 87%. Anal. Calc. C, 43.55; H, 4.62; N, 21.38; Cu, 12.13%. Found: C, 43.21; H, 4.53; N, 21.15; Cu, 12.02%. IR (KBr, cm^−1^): 3436, 1608, 1568 and 1384.

### 3.3. Hirshfeld Surface Analysis

Hirshfeld surface analyses were performed using Crystal Explorer 17.5 software [61].

### 3.4. Biological Studies

The antimicrobial, antioxidant and anticancer activities of the studied compounds are described in Methods S2–S4 (Supplementary material).

## 4. Conclusions

The biological evaluations of the antimicrobial, antioxidant and anticancer activities for two *s*-Triazine Schiff base ligands and their Cu(II) complexes were examined. The structures of the newly synthesized complexes were determined using different analytical and spectroscopic techniques to be [Cu(DMAT)(H_2_O)(NO_3_)]NO_3_*C_2_H_5_OH, [Cu(DMOT)(CH_3_COO)] (**2**) and [Cu(DMOT)(NO_3_)] (**3**). The molecular and supramolecular structures of **1** and **2** were described on the basis of X-ray single-crystal diffraction and Hirshfeld analysis. While complex **1** had a hexa-coordinated CuN3O3 coordination environment, complex **2** had a CuN2O2 tetra-coordinated Cu(II) ion. Complex **1** (IC_50_ = 5.94 ± 0.58 µM) had a four times higher level of anticancer activity against lung carcinoma A-549 than did *cis*-platin (IC_50_ = 25.01 ±2.29 µM). In addition, the selectivity index of **1** (6.34) was the highest, indicating that complex **1** is the best candidate for use as an anticancer agent against the lung carcinoma A-549 cell line. The same complex also had the highest amount of antioxidant activity compared to ascorbic acid as a control. Both the organic ligands had no antimicrobial activity at the applied concentration. In contrast, the Cu(II) complexes showed interesting antimicrobial activity against the studied microbes. The three complexes all evidenced a strong antibacterial activity towards *B. subtilis*.

## Figures and Tables

**Table 1:** The most important bond distances (Å) and angles (˚) of complex **1**.

Bond	Distance	Bond	Distance
Cu1-N1	1.989 (2)	Cu1-O4	2.006 (2)
Cu1-N2	1.990 (2)	Cu1-O1	2.185 (2)
Cu1-N4	2.022 (2)	Cu1-O5	2.651 (2)
**Bonds**	**Angle**	**Bonds**	**Angle**
N1-Cu1-N4	163.73 (9)	O4-Cu1-N4	95.83 (8)
N1-Cu1-O1	92.13 (9)	O4-Cu1-O1	103.61 (9)
N1-Cu1-O4	88.16 (9)	O5-Cu1-O4	53.28 (8)
N2-Cu1-N4	82.26 (8)	O5-Cu1-N1	83.17 (9)
N2-Cu1-O1	98.06 (10)	O5-Cu1-N2	104.87 (9)
N2-Cu1-O4	158.14 (9)	O5-Cu1-N4	86.58 (9)
N4-Cu1-O1	102.19 (9)	O5-Cu1-O1	156.38 (9)
N1-Cu1-N2	88.17 (9)		

**Table 2:** The hydrogen bond parameters of complexes **1** and **2**.

Atoms	D-H (Å)	H…A (Å)	D…A (Å)	D-H…A (º)
N1-H1A...O6 ^i^	0.84 (3)	2.17 (4)	2.975 (4)	161 (3)
N1-H1B...O2 ^ii^	0.85 (3)	2.14 (3)	2.932 (3)	155 (2)
O1-H1C...O7 ^iii^	0.81 (3)	2.01 (3)	2.787 (4)	162 (4)
O1-H1D...O3 ^iv^	0.79 (4)	2.00 (4)	2.767 (4)	163 (4)
N3-H3A...O7	0.84 (3)	2.12 (3)	2.936 (4)	165 (3)
**Symm. Code:** (i) 2-x,1-y,1-z; (ii) -1 + x,y,z; (iii) 2-x,1-y,1-z; (iv) x,1 + y,z				

**Table 3:** The most important bond distances (Å) and angles (˚) for complex **2**.

Bond	Distance	Bond	Distance
Cu1-O3	1.905(7)	Cu2-O10	1.902(8)
Cu1-O1	1.873(6)	Cu2-O9	1.852(7)
Cu1-N2	1.945(8)	Cu2-N10	1.938(8)
Cu1-N4	1.976(7)	Cu2-N12	1.984(8)
**Bonds**	**Angle**	**Bonds**	**Angle**
O3-Cu1-N2	160.9(3)	O10-Cu2-N10	164.1(3)
O3-Cu1-N4	97.4(3)	O10-Cu2-N12	96.2(3)
O1-Cu1-O3	93.0(3)	O9-Cu2-O10	92.5(3)
O1-Cu1-N2	93.2(3)	O9-Cu2-N10	92.5(4)
O1-Cu1-N4	160.2(3)	O9-Cu2-N12	163.9(3)
N2-Cu1-N4	82.4(3)	N10-Cu2-N12	82.1(4)

**Table 4:** The short-distance interactions and their distances.

Contact	Distance	Contact	Distance
1		2	
O5…H14A	2.601	O8…H3A	2.397
O2…H1B	1.996	O4…H11	1.857
O7…H1C	1.841	O2…H3	1.802
O8…H1C	2.543	O5…H24	2.483
O3…H1D	1.820	O7…H40A	2.584
O8…H19A	2.577	O6…H16B	2.551
O6…H2A	2.419	O6…H35B	2.361
O6…H1A	2.008	O7…H21B	2.602
O7…H3A	1.955	C22…H11B	2.547
O7…H11C	2.593	C1…H32B	2.713
O9…H11C	2.509	C10…H39B	2.762
O9…H3A	2.483	C21…H39B	2.710
C11…C11	3.350	N14…H14B	2.490
H4…H4	2.074	C1…C7	3.332
		C6…C6	3.399

**Table 5:** Inhibition zone diameters (mm) for the tested microbes ^a^.

Microbe	Type	DMAT	DMOHT	1	2	3	Control
*A. fumigatus*	Fungi	NA *	NA *	14	15	NA	17 ^a^
*C. albicans*		NA *	NA *	NA	10	NA	20 ^a^
*S. aureus*	Gram positive bacteria	NA *	NA *	10	11	12	24 ^b^
*B. subtilis*		NA *	NA *	25	25	24	26 ^b^
*E. coli*	Gram negative bacteria	NA *	NA *	11	14	15	30 ^b^
*P. vulgaris*		NA *	NA *	15	15	13	25 ^b^

^a^ Ketoconazole and ^b^ Gentamycin, * NA: Not active.

**Table 6:** MIC values (μg/mL) for the studied compounds.

Microbe	1	2	3	Control
*A. fumigatus*	1250	625	ND *	156.25 ^a^
*C. albicans*	ND*	5000	ND *	312.5 ^a^
*S. aureus*	2500	1250	1250	9.7 ^b^
*B. subtilis*	39.06	39.06	78.125	4.8 ^b^
*E. coli*	2500	1250	312.5	4.8 ^b^
*P. vulgaris*	625	1250	1250	4.8 ^b^

* ND: Not determined ^a^ Ketoconazole ^b^ Gentamycin.

**Table 7:** The antioxidant activity of the studied compounds.

Conc. (μg/mL)		DPPH Scavenging %				
	Ascorbic Acid	DMAT	DMOHT	1	2	3
1280	98.91	95.31	93.87	96.84	94.54	95.76
640	97.83	94.08	88.54	95.07	92.15	94.29
320	95.64	91.37	84.93	92.48	88.03	90.81
160	92.31	86.59	73.64	87.65	83.14	85.92
80	90.25	79.28	61.32	82.31	78.62	81.47
40	83.09	70.89	47.09	77.49	67.84	68.9
20	71.38	54.23	31.95	68.61	49.08	51.32
10	48.52	35.17	19.87	40.93	36.52	32.95
5	40.36	28.65	10.48	27.86	17.66	18.73
2.5	34.57	20.79	5.73	21.32	9.39	11.46
IC_50_	10.62 ± 0.84	17.83 ± 0.65	48.2 ± 1.08	13.34 ± 0.58	20.93 ± 0.91	19.31 ± 0.73

**Table 8:** IC_50_, CC_50_ and selectivity index (SI) values (μM) for the studied compounds.

	DMAT	DMOHT	Complex 1	Complex 2	Complex 3	*cis*-Platin
**IC_50_**	551.48	564.51	5.94	141.47	58.34	25.01
**CC_50_**	716.04	999.90	37.67	101.47	172.96	242.25
**SI = CC_50_/IC_50_**	1.30	1.77	6.34	0.72	2.96	9.69

## Data Availability

Not applicable.

## Supplementary Materials

The following supporting information can be downloaded at https://www.mdpi.com/article/10.3390/molecules27092989/s1, Materials and physical measurements, X-ray photoelectron spectroscopy (XPS) measurement, Single-crystal X-ray diffraction analysis and structure determination, Figure S1: ^1^H and ^13^C NMR spectra of **DMAT**, Figure S2:^1^H and ^13^C NMR spectra of **DMOHT**, Figure S3: FTIR spectra of the free **DMAT** and complex **1**, Figure S4: FTIR spectra of the free **DMOHT**, complexes **2** and **3**, Figure S5: XPS spectra for **DMAT**, **DMOHT**, Complexes **1**, **2** and **3**, Table S1: Crystal data and refinement details of the studied complexes, Table S2: The percentages of different contacts in the crystal structure of complexes **1** and **2**, Table S3: XPS analysis of **DMAT** and complex **1**, Table S4: XPS analysis of **DMOHT**, complexes **2** and **3**, Table S5: Inhibitory activity against Lung carcinoma A-549 cells for **DMAT**, Table S6: Inhibitory activity against Lung carcinoma A-549 cells for **DMAT**, Table S7: Inhibitory activity against Lung carcinoma A-549 cells for **DMOHT**, Table S8: Inhibitory activity against Lung carcinoma A-549 cells for **2**, Table S9: Inhibitory activity against Lung carcinoma A-549 cells for **DMAT**, Table S10: Inhibitory activity against Lung carcinoma MRC-5 cells for **DMAT**, Table S11: Inhibitory activity against Lung carcinoma MRC-5 cells for **1**, Table S12: Inhibitory activity against Lung carcinoma MRC-5 cells for **DMOHT**, Table S13: Inhibitory activity against Lung carcinoma MRC-5 cells for **2**, Table S14: Inhibitory activity against Lung carcinoma cells for **3**, Method S1: Preparation of the studied ligands, Method S2: Antimicrobial studies, Method S3: DPPH Radical Scavenging Activity, Method S4: Evaluation of Cytotoxic activity.

## Sample Availability

Samples of the compounds are not available from the authors.
