# Supplementary material for: Synthesis, X-ray Structure and Biological Studies of New Self-Assembled Cu(II) Complexes Derived from s-Triazine Schiff Base Ligand
Source: Molecules. 2022 May 6;27(9):2989. doi: 10.3390/molecules27092989 (PMC9106035; doi:10.3390/molecules27092989)
Supplement: Supplementary file 1 [file molecules-27-02989-s001.zip › Supplementary Materials.pdf]

Synthesis, X-ray structure and biological studies of new self-assembled Cu(II) complexes derived from *s*-triazine Schiff base ligand

Tarek E. Khalil[1]*, Kholood A. Dahlous[2]*, Saied M. Soliman[1]*, Nessma A. Khalil[1], Ayman El-Faham[1], and Ali El-Dissouky[1]

[1] Department of Chemistry, Faculty of Science, Alexandria University, P.O. Box 426, Ibrahimia, Alexandria 21321, Egypt. Emails: tarekakel@alexu.edu.eg (T.E.K), saied1soliman@yahoo.com (S.M.S), nessma_1988@outlook.com (N.A.K), alieldissouky@alexu.edu.eg (A.E.-D) and aymanel_faham@hotmail.com (A.E-F).

[2] Department of Chemistry, College of Science, King Saud University, P. O. Box 2455, Riyadh 11451, Saudi Arabia. Email: kdahloos@ksu.edu.sa (Kh.A.D).

* Correspondence: Tarek E. Khalil tarekakel@alexu.edu.eg (T.E.K), Saied M. Soliman: saied1soliman@yahoo.com (S.M.S) and Kholood A. Dahlous: kdahloos@ksu.edu.sa (Kh.A.D)

Materials and physical measurements

Chemicals were purchased from Sigma-Aldrich Company (Chemie GmbH, 82024 Taufkirchen, Germany). Elemental analyses (carbon, hydrogen and nitrogen) were performed on a Perkin-Elmer 2400 elemental analyzer (PerkinElmer Inc., Waltham, MA USA). Copper content was determined using Shimadzu atomic absorption spectrophotometer (Shimadzu AA-7000 series). ^{1}H and ^{13}C NMR spectra were recorded on 500 MHz JEOL spectrometer (JEOL Ltd., Tokyo, Japan) at room temperature in $CDCl_3$ as solvent. Chemical shifts were reported in parts per million (ppm).

X-ray photoelectron spectroscopy (XPS) measurement

X-ray photoelectron spectroscopy (XPS) analyses were performed by K-ALPHA (Thermo Fisher Scientific, USA) with monochromatic X-ray Al K-alpha radiation (energy -10 to 1350 eV), under a vacuum of 10^{-9} mbar with full-spectrum pass energy 200 eV at narrow-spectrum 50 eV. The analysis spot size was 400 µm in diameter. All binding energy values were determined concerning C1s line originating from adventitious carbon. The instrument is located at Central Metallurgical Research, Institute (CMRDI), Al Tbin, Cairo, Egypt.

Single crystal X-ray diffraction analysis and structure determination

The crystallographic measurement of the Cu(II) complexes was performed using CrysAlis CCD, Oxford Diffraction limited, version 1.70.14 (2002), using graphite monochromated MoKα radiation (λ = 0.71013 Å) at Technische Universitaet Darmstadt, FB Materialwissenschaft, FG Strukturforschung, Darmstadt, Germany. The structures were solved by direct method with SHELXS–97; refinement was done by full–matrix least squares on F^2 using the SHEXL–97 [S1]. Hydrogen atoms were positioned with idealized geometry using a riding model and were refined with isotropic displacement parameter.

[S1] G.M. Sheldrick, *Acta Cryst.*, **2008**, *A64*, 112-122. https://doi.org/10.1107/S0108767307043930.

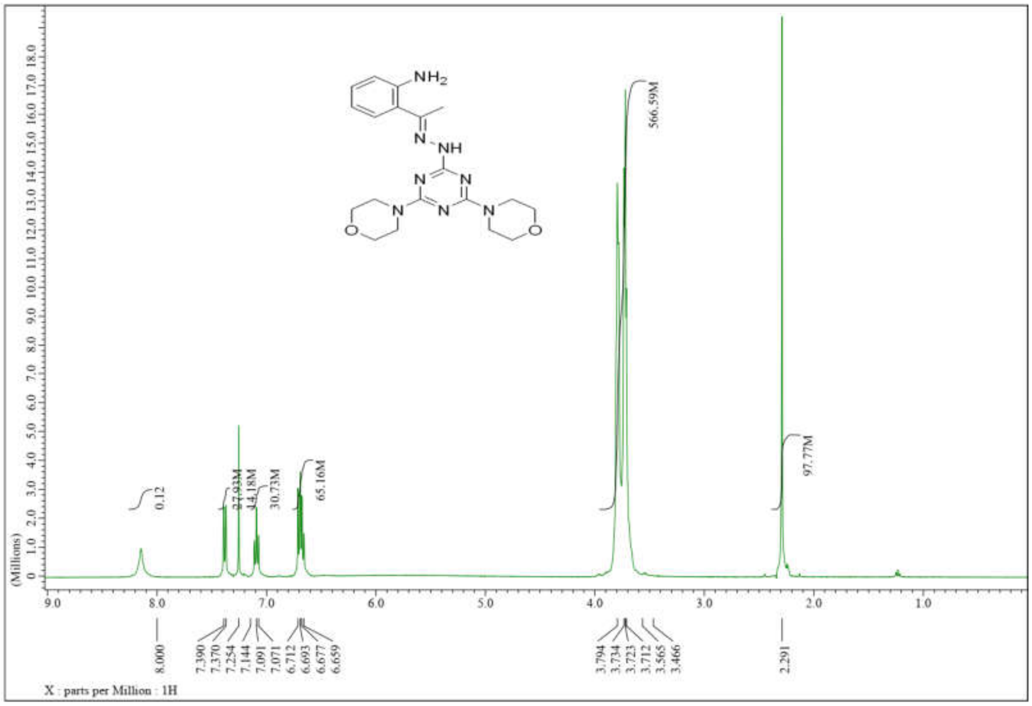

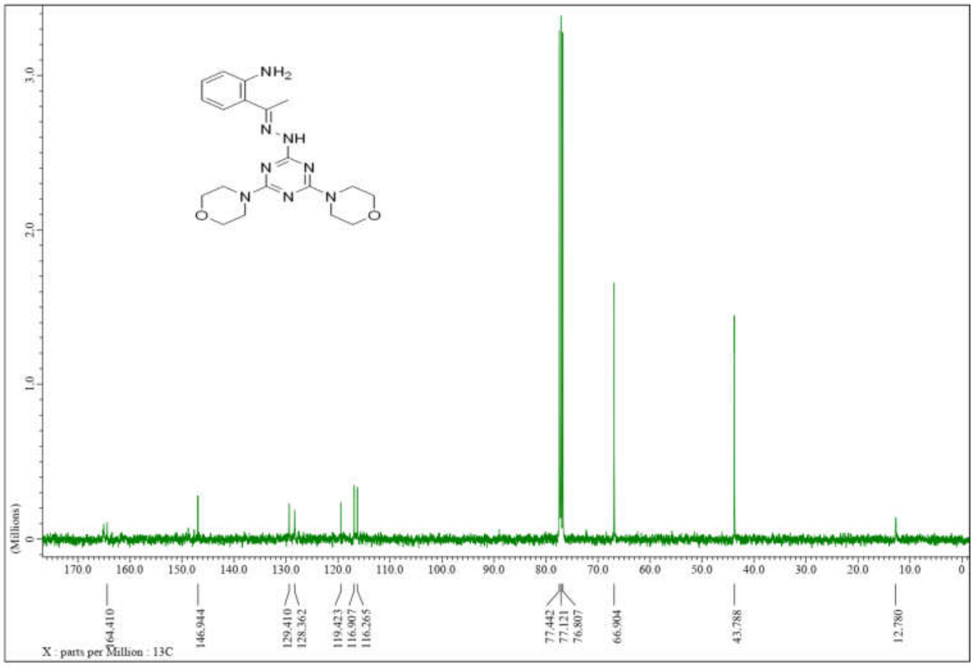

Figure S1 ^{1}H and ^{13}C NMR spectra of **DMAT**.

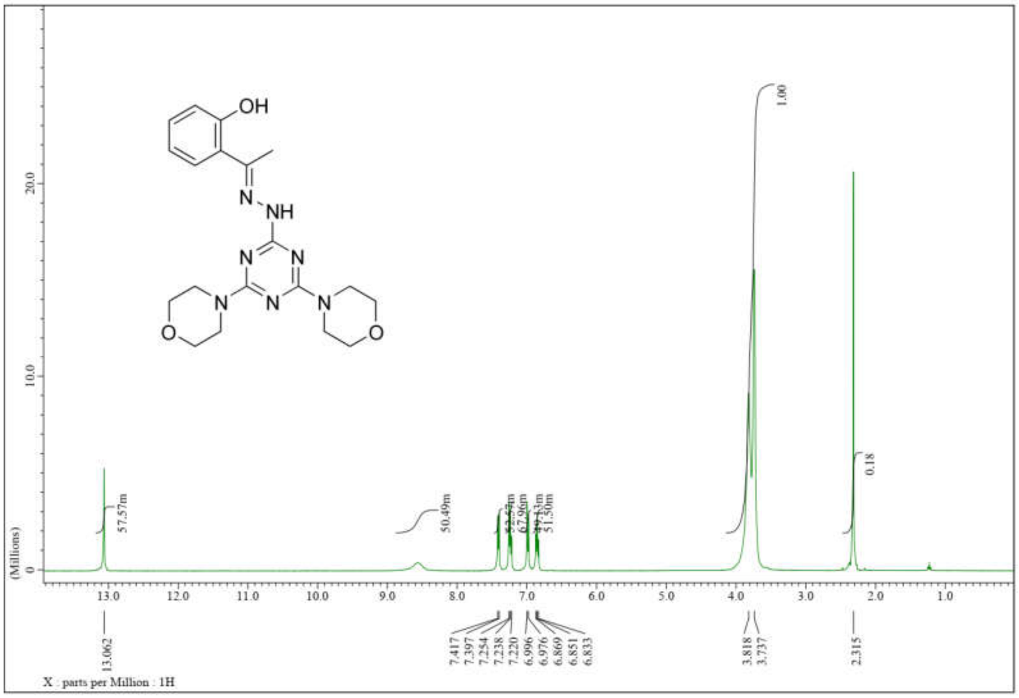

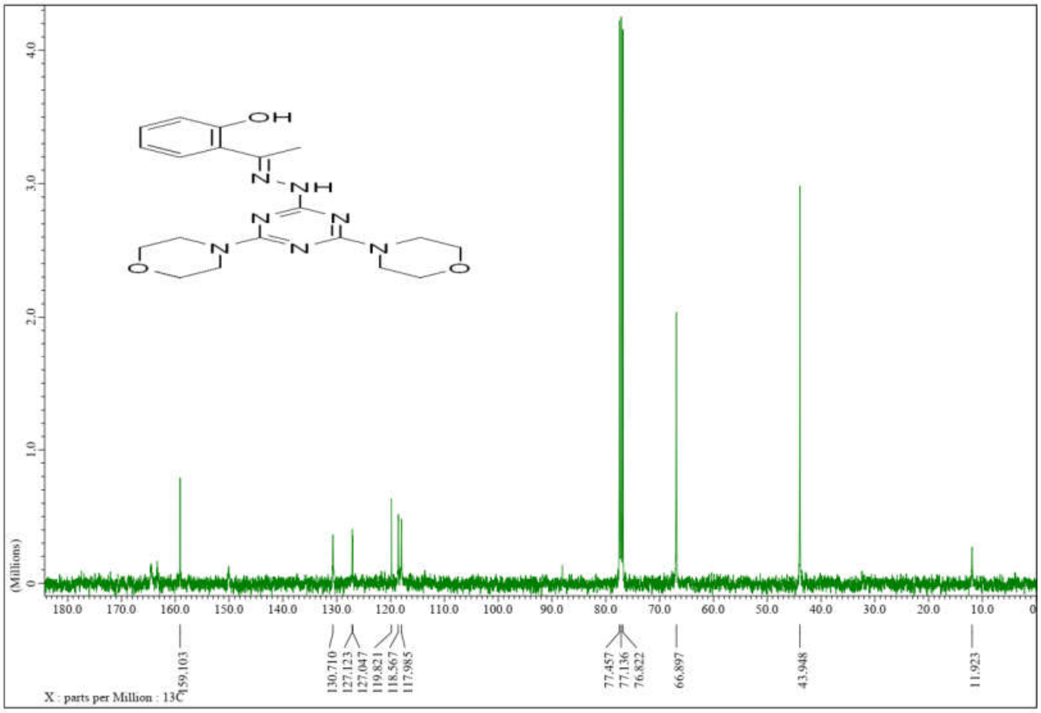

Figure S2. ^{1}H and ^{13}C NMR spectra of **DMOHT**.

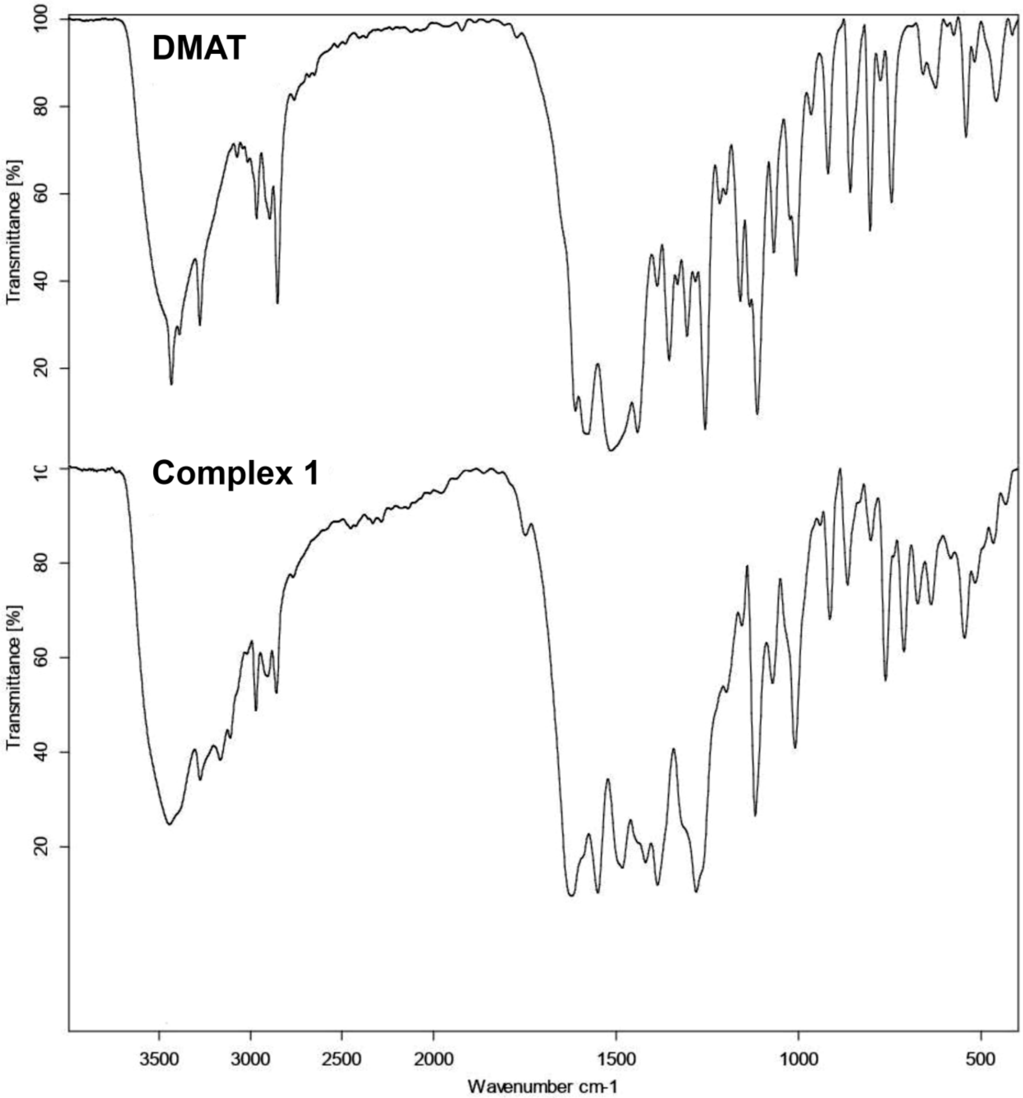

Figure S3 FTIR spectra of the free **DMAT** and complex **1**.

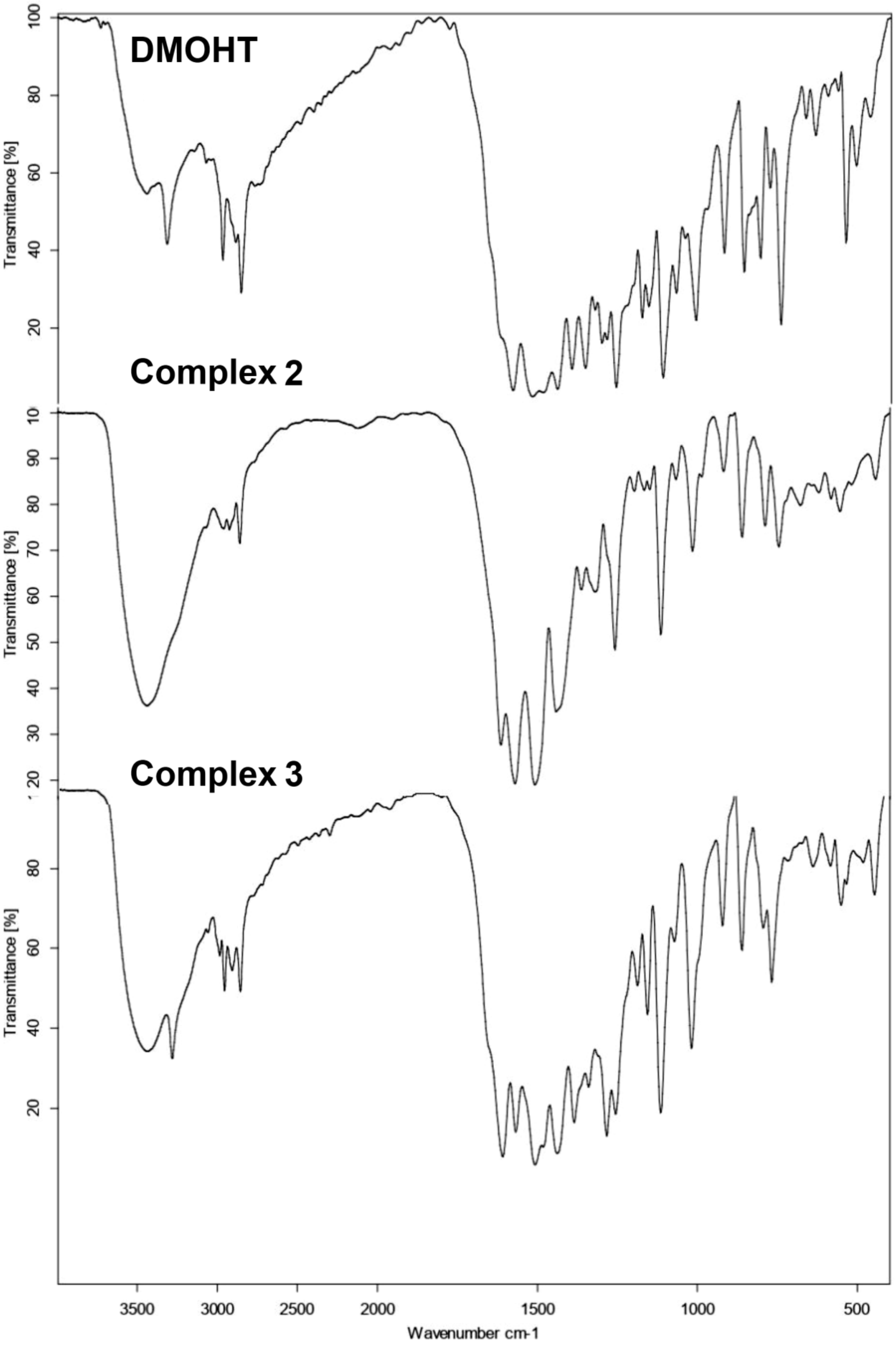

Figure S4 FTIR spectra of the free **DMOHT**, complexes **2** and **3**.

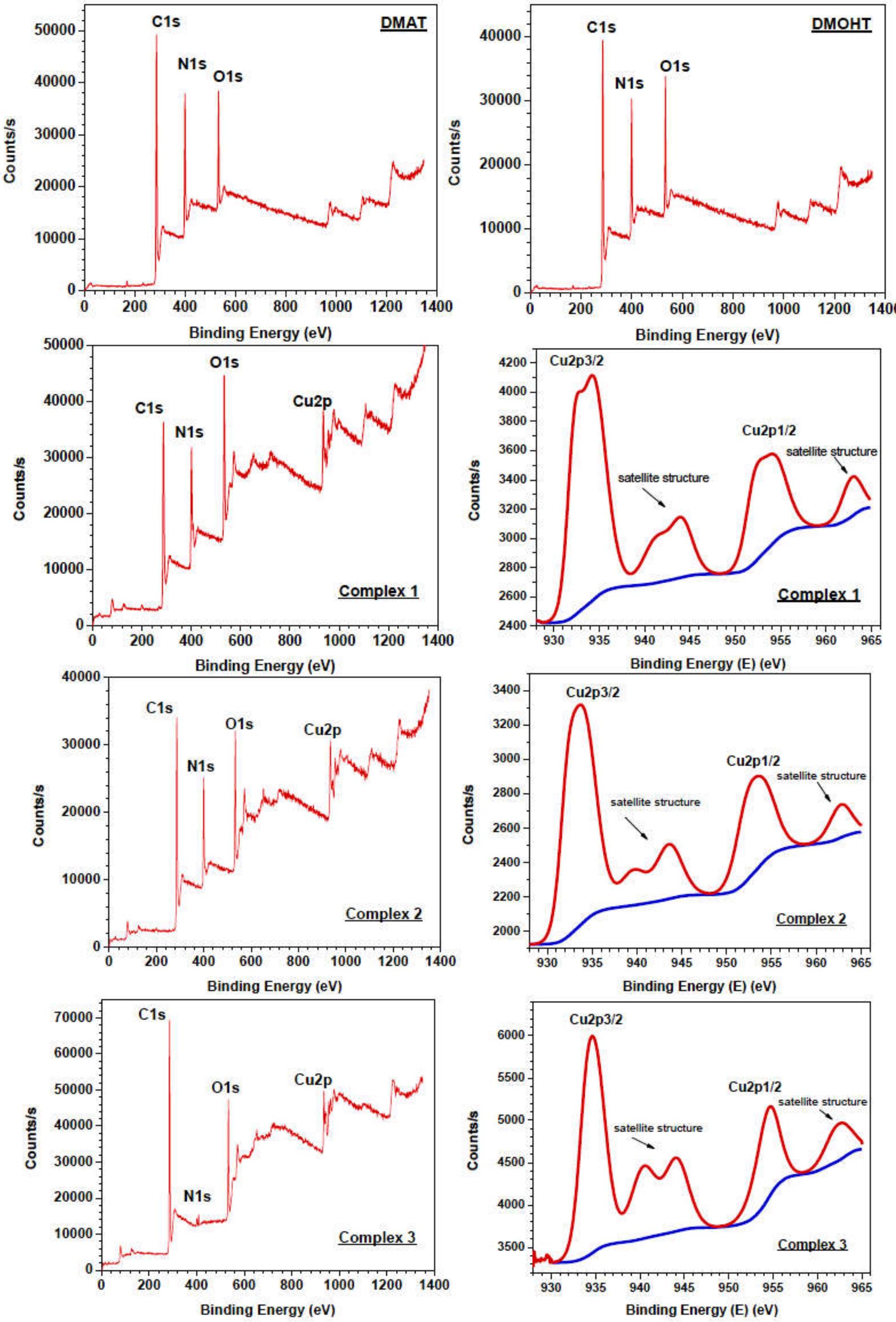

Figure S5 XPS spectra for **DMAT**, **DMOHT**, Complexes **1**, **2** and **3**.

Table S1 Crystal data and refinement details of the studied complexes.

Compound	**1**	**2**
Empirical formula	$C_{21}H_{34}CuN_{10}O_{10}$	$C_{42}H_{54}Cu_2N_{14}O_{10}$
Formula weight	650.12	1042.07
Temperature/K	293(2)	293(2)
Crystal system	Triclinic	Monoclinic
Space group	P-1	$P2_1/n$
a/Å	9.1125(4)	14.4540(10)
b/Å	12.6280(7)	29.535(2)
c/Å	13.7805(8)	14.7520(10)
α/°	116.552(6)	90
β/°	93.066(4)	114.450(10)
γ/°	90.981(4)	90
Volume/Å^3	1414.98(15)	5732.9(8)
Z	2	4
ρ_{calc}g/cm^3	1.526	1.207
μ/mm^{-1}	0.843	0.801
F(000)	626	2168
Crystal size/mm^3	0.4 × 0.28 × 0.22	0.34 × 0.2 × 0.08
Radiation	MoKα (λ = 0.71073)	MoKα (λ = 0.71073)
2Θ range for data collection/°	5.624 to 50.698	5.13 to 50.698
Index ranges	-8 ≤ h ≤ 10, -15 ≤ k ≤ 14, -16 ≤ l ≤ 16	-17 ≤ h ≤ 10, -34 ≤ k ≤ 35, -13 ≤ l ≤ 17
Reflections collected	9252	22871
Independent reflections	5096 [R_{int} = 0.0178, R_{sigma} = 0.0260]	10300 [R_{int} = 0.1712, R_{sigma} = 0.3966]
Data/restraints/parameters	5096/5/368	10300/21/617
Goodness-of-fit on F^2	1.024	0.886
Final R indexes [I>=2σ (I)]	R_1 = 0.0388, wR_2 = 0.0931	R_1 = 0.0943, wR_2 = 0.1504
Final R indexes [all data]	R_1 = 0.0508, wR_2 = 0.1014	R_1 = 0.2891, wR_2 = 0.2087
Largest diff. peak/hole / e Å^{-3}	0.49/-0.66	0.45/-0.39
**CCDC**	**2164465**	**2164466**

Table S2 The percentages of different contacts in the crystal structure of complexes **1** and **2**.

Contact	1	2A	2B
Cu…N		0.1	0.1
Cu…H		0.6	0.7
Cu…C		0.3	
N…O	1.1	0.7	0.7
O…O	1.0		
N…N	0.9	1.4	1.3
N…H	4.3	5.1	4.8
C…N	0.5	2.3	1.1
C…O	0.1	0.8	0.8
O…H	37.2	22.0	22.5
C…C	0.1	2.0	2.7
C…H	11.8	8.6	8.8
H…H	43.0	56.1	56.5

Table S3 XPS analysis of **DMAT** and complex **1.**

**Peak (Core Level)**	**BE/eV**	**%**	**FWHM (eV)**	**Peak (Core Level)**	**BE/eV**	**%**	**Δ(BE) /eV Doublet separation**	**FWHM (eV)**
**DMAT**						**Complex 1**		
C1s	286.17	66.46	4.02	C1s	287.22	58.95		5.07
N1s	399.75	21.46	3.87	N1s	400.85	19.54		4.64
O1s	532.94	11.14	3.5	O1s	533.74	17.54		4.45
C1s	285.74	18.98	1.22	C1s	285.66	41.6		1.75
C1s A	287.05	23.54	1.93	C1s A	287.37	14.94		1.69
C1s B	284.71	54.77	2.17	C1s B	284.49	43.46		1.9
C1s C	289.37	2.71	3.37					
N1s B	399.45	61	2.14	N1s	399.58	28.68		1.77
N1s D	398.01	39	1.59	N1s A	406.09	14.55		1.74
				N1s B	398.23	37.59		1.92
				N1s C	399.91	19.18		2.12
O1s	532.31	55.66	1.58	O1s	532	71.15		1.71
O1s A	530.85	18.29	1.79	O1s A	531.37	20.44		2.66
O1s B	532.78	26.04	2.77	O1s B	532.99	8.41		1.69
				Cu2p, 3/2	934.28	41.38		3.37
				Cu2p, ½	954.23	15.78	**19.95**	3.37

Table S4 XPS analysis of **DMOHT**, complexes **2** and **3.**

**Peak**	**BE/eV**	**%**	**FWHM**	**Peak (Core**	**BE/eV**	**%**	**FWHM**	**Δ(BE) /eV**	**Peak (Core**	**BE/eV**	**%**	**FWHM**	**Δ(BE) /eV**
	**DMOHT**					**Complex 2**					**Complex 3**		
C1s	286.24	66.44	4.13	C1s	286.07	65.08	4.39		C1s	285.91	3.25	78.64	
N1s	399.94	20.37	3.86	N1s	399.87	17.3	3.67		N1s	407.85	2.59	1.46	
O1s	533.13	12.46	3.4	O1s	532.82	14.37	3.64		O1s	533.16	3.45	15.98	
C1s A	284.42	39.73	1.77	C1s A	286.67	40.07	3.07		C1s	284.9	55.56	1.42	
C1s B	287.31	19.45	1.78	C1s B	285.66	19.9	1.4		C1s A	285.26	38.46	2.57	
C1s C	285.85	40.82	1.45	C1s C	284.32	40.03	1.92		C1s B	288.76	5.98	1.64	
N1s	400.84	7.91	1.83	N1s	400.49	0.42	0.49		N1s	407.22	55.33	1.81	
N1s A	398.34	51.9	1.82	N1s A	397.88	6.97	1.13		N1s A	400.26	19.93	1.68	
N1s B	399.79	40.2	1.46	N1s B	399.18	92.61	2.52		N1s B	399.01	24.74	2.15	
O1s	532.47	59.97	1.61	O1s	532.75	27.43	1.24		O1s	531.96	18.89	1.53	
O1s A	532.75	20.56	3.07	O1s A	531.9	39.13	1.36		O1s A	533.35	3.15	1.47	
O1s B	531.39	19.47	2.36	O1s B	530.2	9.59	1.51		O1s C	532.47	77.95	2.38	
O1s C				O1s C	530.87	16.54	1.4						
O1s D				O1s D	533.84	7.3	1.26						
				Cu2p, 3/2	932.25	10.95	1.97		Cu2p, 3/2	934.72	37.77	3.28	
				Cu2p, 1/2	952.14	10.33	2.85	**19.89**	Cu2p, 1/2	954.71	11.78	2.61	**19.99**

Table S5 Inhibitory activity against Lung carcinoma A-549 cells for **DMAT**.

Sample conc. (μM)	Viability %	Inhibitory %	S.D. (±)
1254.8	17.64	82.36	3.12
627.4	39.56	60.44	2.08
313.7	82.71	17.29	1.93
156.8	98.04	1.96	0.62
78.4	100	0	
39.1	100	0	
19.6	100	0	
9.8	100	0	
5.0	100	0	
2.5	100	0	
0.0	100	0	

Table S6 Inhibitory activity against Lung carcinoma A-549 cells for **1**.

Sample conc. (μM)	Viability %	Inhibitory %	S.D. (±)
769.1	0.89	99.11	0.17
384.5	2.97	97.03	0.31
192.3	6.54	93.46	0.32
96.1	11.28	88.72	0.46
48.1	19.46	80.54	1.02
24.0	30.95	69.05	0.97
12.0	42.78	57.22	1.46
6.0	49.21	50.79	1.73
3.1	60.73	39.27	2.31
1.5	68.94	31.06	0.72
0.0	100	0	

Table S7 Inhibitory activity against Lung carcinoma A-549 cells for **DMOHT**.

Sample conc. (μM)	Viability %	Inhibitory %	S.D. (±)
1251.7	21.54	78.46	1.72
625.8	43.91	56.09	2.85
312.9	75.08	24.92	3.14
156.5	90.67	9.33	1.21
78.2	98.25	1.75	0.79
39.1	100	0	
19.5	100	0	
9.8	100	0	
5.0	100	0	
2.5	100	0	
0.0	100	0	

Table S8 Inhibitory activity against Lung carcinoma A-549 cells for **2**.

Sample conc. (μM)	Viability %	Inhibitory %	S.D. (±)
959.6	3.62	96.38	0.44
479.8	7.85	92.15	0.37
239.9	16.43	83.57	0.59
120.0	28.70	71.3	0.62
60.0	41.37	58.63	0.97
29.9	59.43	40.57	2.09
15.0	78.12	21.88	1.46
7.5	89.41	10.59	0.63
3.8	97.16	2.84	0.58
1.9	100	0	
0.0	100	0	

Table S9 Inhibitory activity against Lung carcinoma A-549 cells for **3**.

Sample conc. (μM)	Viability %	Inhibitory %	S.D. (±)
954.2	4.87	95.13	0.45
477.1	11.28	88.72	0.64
238.6	23.94	76.06	1.32
119.3	36.62	63.38	1.06
59.6	49.28	50.72	2.54
29.8	67.19	32.81	2.37
14.9	80.46	19.54	1.28
7.4	92.75	7.25	0.91
3.8	98.63	1.37	0.59
1.9	100	0	
0.0	100	0	

Table S10 Inhibitory activity against Lung carcinoma MRC-5 cells for **DMAT**.

Sample conc. (μM)	Viability %	Inhibitory %	S.D. (±)
1254.8	26.06	73.94	3.72
627.4	53.94	46.06	4.08
313.7	89.27	10.73	2.19
156.8	99.85	0.15	0.43
78.4	100	0	
39.1	100	0	
19.6	100	0	
9.8	100	0	
5.0	100	0	
2.5	100	0	
0.0	100	0	

Table S11 Inhibitory activity against Lung carcinoma MRC-5 cells for **1**.

Sample conc. (μM)	Viability %	Inhibitory %	S.D. (±)
769.1	1.93	98.07	0.41
384.5	5.72	94.28	0.26
192.3	10.89	89.11	0.75
96.1	23.71	76.29	1.09
48.1	40.67	59.33	2.45
24.0	62.39	37.61	3.17
12.0	74.85	25.15	2.11
6.0	89.13	10.87	0.95
3.1	96.28	3.72	0.64
1.5	99.41	0.59	0.37
0.0	100	0	

Table S12 Inhibitory activity against Lung carcinoma MRC-5 cells for **DMOHT**.

Sample conc. (μM)	Viability %	Inhibitory %	S.D. (±)
1251.7	38.19	61.81	3.43
625.8	67.56	32.44	2.82
312.9	89.62	10.38	1.46
156.5	97.86	2.14	0.92
78.2	100	0	
39.1	100	0	
19.5	100	0	
9.8	100	0	
5.0	100	0	
2.5	100	0	
0.0	100	0	

Table S13 Inhibitory activity against Lung carcinoma MRC-5 cells for **2**.

Sample conc. (μM)	Viability %	Inhibitory %	S.D. (±)
959.6	5.74	94.26	0.62
479.8	15.21	84.79	1.73
239.9	27.98	72.02	2.84
120.0	42.35	57.65	3.13
60.0	67.18	32.82	2.86
29.9	86.24	13.76	2.12
15.0	92.87	7.13	0.91
7.5	98.71	1.29	0.63
3.8	100	0	
1.9	100	0	
0.0	100	0	

Table S14 Inhibitory activity against Lung carcinoma cells for **3**.

Sample conc. (μM)	Viability %	Inhibitory %	S.D. (±)
954.2	7.54	92.46	0.68
477.1	20.65	79.35	2.03
238.6	41.28	58.72	1.76
119.3	57.14	42.86	2.02
59.6	76.92	23.08	2.14
29.8	89.17	10.83	1.29
14.9	98.63	1.37	0.71
7.4	100	0	
3.8	100	0	
1.9	100	0	
0.0	100	0	

Method S1 Preparation of the studied ligands

Preparation of 2-hydrazino-4,6-dimorpholino-1,3,5-triazine

Hydrazine hydrate 80% (10 mL) were added dropwise to a solution of 2-chloro-4,6-disubstituted-1,3,5-triazine (20 mmol) in 50 mL ethanol and then the reaction mixture was refluxed for 4-6h. After that the solvent was removed under vacuum and excess ether was added to afford the product as a white solid in yield > 90% and used directly in the next step.

Preparation of the ligands DMAT and DMOHT

2-Hydrazino-4,6-dimorpholino-1,3,5-triazine (10 mmol) was added to a solution of 2-substituted acetophenone (10 mmol) in ethanol (30 mL) containing 2-3 drops of acetic acid, and the reaction mixture was stirred with gentle reflux for 4 h. The solvent was concentrated under vacuum and the precipitated product was filtered off and dried at room temperature. The product was collected and recrystallized from ethylacetate to afford the products in pure state.

2-(1-(2-(4,6-dimorpholino-1,3,5-triazin-2-yl)hydrazono)ethyl)aniline ***(DMAT)***

Pale yellow solid in yield 76%; mp199-201°C; IR (KBr): 3427 (NH), 3268 (NH),1650 (C=N), 1519 (C=N), 1438 (C=C) cm^{-1}; ^1H NMR ($CDCl_3$) δ: 2.29 (3H, s, CH_3), 3.71-3.72 (8H, m, 4 CH_2), 3.78-3.79 (8H, m, 4 CH_2), 6.66-6.71 (2H, m, Ar), 7.09(1H, t, *J* = 8.0, Ar), 7.38 (1H, d, *J* = 8.0, 1HAr), 8.15 (1H, s, NH); ^{13}C NMR ($CDCl_3$) δ: 12.8, 43.8, 66.9, 116.3, 116.9, 119.4, 128.4, 129.4, 146.9, 164.4, 165.4.

2-(1-(2-(4,6-dimorpholino-1,3,5-triazin-2-yl)hydrazono)ethyl)phenol *(**DMOHT**)*

White solid in yield 75%; mp 225-228 °C; IR (KBr): 3420 (NH), 3313 (OH), 1579 (C=N), 1514 (C=N), 1438 (C=C) cm^{-1}; ^1H NMR ($CDCl_3$) δ: 2.37 (3H, s, CH_3), 3.74-3.82 (16H, m, 8 CH_2), 6.85 (1H, t, *J* = 15.2, Ar), 6.98(1H, d, *J* = 8.0, Ar), 7.25 (1H, d, *J* = 7.2, Ar), 7.40 (1H, d, *J* = 8.0, Ar), 8.56 (1H, brs, NH), 13.06 (1H, s, OH); ^{13}C NMR ($CDCl_3$) δ: 11.9, 43.9, 66.9, 117.9, 118.1, 119.8, 127.1, 130.7, 159.1, 163.4, 164.4.

Method S2: Antimicrobial studies

a) Tested pathogenic microbes

The antibacterial activity of the studied ligands and their Cu(II) complexes were evaluated against two Gram positive bacteria ((*S. aureus* (ATCC 25923) and *B. subtilis* (RCMB015(1)NRR LB-543)), two Gram negatvie bacteria ((*E. coli* (ATCC 25922) and *P. vulgaris* (RCMB 004(1)ATCC 13315)) and two fungi ((*A. fumigatus* (RCMB 002008) and *C. albicans* (RCMB 005003(1) ATCC 10231)). Gentamycin was used as standard antibacterial agent. The samples maintained in Brain heart infusion (BHI) at 20ºC; 300 mL of each stock–culture was added to 3 mL of BHI broth. Overnight cultures were kept for 24 h at 37 ºC ± 1ºC and the purity of cultures was checked after 24 h of incubation. After 24 h of incubation, bacterial suspension was diluted with sterile physiological solution, for the diffusion and indirect bioautographic tests, to 108 CFU/mL (turbidity = McFarland barium sulfate standard 0.5). In case of fungi *A. fumigatus* (RCMB 002008) and *C. albicans* (RCMB 005003(1) ATCC 10231), the used medium in antagonistic activity against tested fungi is Potato Dextrose Agar, where Fluconazole was used standard antifungal agent.

b) Agar well diffusion method

Synthetic compounds were prepared at concentration 10 mg/mL dissolved in DMSO as stock solutions. Preparation of sterilized Mueller Hinton agar plates seeded with tested pathogenic bacteria occurred. The wells are done by sterilized cork borer in size 6 mm and hence 200 µg of the synthetic compound was poured in each well comparably with DMSO as control. The plates were incubated at 37°C for 24 h. after incubation period; antimicrobial activity was determined by inhibition zones.

c) Minimum Inhibitory Concentration (MIC)

Different dilutions of the compounds are inoculated with tested pathogenic microbes. After incubation period of 96 well microplate, the results are measured using microplate reader. To determine at what level the MIC endpoint is established; subculture of test samples at different concentrations occurred in nutrient agar plates.

Method S3 DPPH Radical Scavenging Activity:

Freshly prepared (0.004%w/v) methanol solution of 2,2-diphenyl-1-picrylhydrazyl (DPPH) radical was prepared and stored at 10 ºC in dark place. A methanolic solution of the test compound was prepared using the same procedure. A 40 uL aliquot of the methanol solution was added to 3mL of DPPH solution. Absorbance measurements were recorded immediately with a UV-visible spectrophotometer (Milton Roy, Spectronic 1201). The decrease in absorbance at 515 nm was determined continuously, with data being recorded at 1 min intervals until the absorbance stabilized (16 min). The absorbance of the DPPH radical without antioxidant (control) and the reference compound ascorbic acid were also measured. All the determinations were performed in three replicates and averaged. The percentage inhibition (PI) of the DPPH radical was calculated according to the formula:

$$PI = [\{(AC - AT)/ AC\} \times 100] \quad (1)$$

Where AC = Absorbance of the control at t = 0 min and AT = absorbance of the sample+DPPH at t = 16 min**.** The 50% inhibitory concentration (IC_{50}), the concentration required to inhibit DPPH radical by 50%, was estimated from graphic plots of the dose response curve.

Method S4 Evaluation of Cytotoxic activity

Cell line Propagation

The cells were propagated in Dulbecco's modified Eagle's medium (DMEM) supplemented with 10% heat-inactivated fetal bovine serum, 1% L-glutamine, HEPES buffer and 50µg/mL Gentamycin. All cells were maintained at 37ºC in a humidified atmosphere with 5% CO_2 and were subcultured two times a week.

Cytotoxicity evaluation using viability assay

For cytotoxicity assay, the cells were seeded in 96-well plate at a cell concentration of 1×10^4 cells per well in 100µl of growth medium. Fresh medium containing different concentrations of the test sample was added after 24 h of seeding. Serial two-fold dilutions of the tested chemical compound were added to confluent cell monolayers dispensed into 96-well, flat-bottomed microtiter plates (Falcon, NJ, USA) using a multichannel pipette. The microtiter plates were incubated at 37ºC in a humidified incubator with 5% CO_2 for a period of 24 h. Three wells were used for each concentration of the test sample. Control cells were incubated without test sample and with or without DMSO. The little percentage of DMSO present in the wells (maximal 0.1%) was found not to affect the experiment. After incubation of the cells for at 37°C, for 24 h, the viable cells yield was determined by the MTT test. Briefly, the media was removed from the 96 well plate and replaced with 100 µL of fresh culture DMEM medium without phenol red then 10 µL of the 12 mM MTT stock solution (5 mg of MTT in 1 mL of PBS) to each well including the untreated controls. The 96 well plates were then incubated at 37°C and 5% CO_2 for 4 hours. An 85 µL aliquot of the media was removed from the wells, and 50 µL of DMSO was added to each well and mixed thoroughly with the pipette and incubated at 37°C for 10 min. Then, the optical density was measured at 590 nm with the microplate reader (SunRise, TECAN, Inc, USA) to determine the number of viable cells and the percentage of viability was calculated as [(ODt/ODc)]x100% where ODt is the mean optical density of wells treated with the tested sample and ODc is the mean optical density of untreated cells. The relation between surviving cells and drug concentration is plotted to get the survival curve of each tumor cell line after treatment with the specified compound. The 50% inhibitory concentration (IC_{50}), the concentration required to cause toxic effects in 50% of intact cells, was estimated from graphic plots of the dose response curve for each conc. using Graphpad Prism software (San Diego, CA. USA).
